# Association of Sarcopenia and Bone Mineral Density in Patients with Chronic Liver Disease: A Prospective Cohort Study Using Standardised Scales

**DOI:** 10.14789/ejmj.JMJ25-0045-OA

**Published:** 2026-01-23

**Authors:** YASHAR MASHAYEKHI, MOHAMED ONSA, KAMRAN KHAN, MUHAMMAD MAHAD KHAN, HAMMAD WASIM, MOHAMMAD GHAITH HULO, MARYAM ZAKA, KHIN MYAT HLA, IBTISSAM WAEL SAAD, SARA LIBA CHANGAAI MANGALOTE, MUSTAFA SARMAD AL HAMDANI, ELWALI ELSHAIKH MOHAMED

**Affiliations:** 1 Department of Orthopaedics, Leicester University Hospitals, Leicester, United Kingdom; 1 Department of Orthopaedics, Leicester University Hospitals, Leicester, United Kingdom; 2 Department of Medicine, Poonch Medical College, Azad Jammu and Kashmir, Pakistan; 2 Department of Medicine, Poonch Medical College, Azad Jammu and Kashmir, Pakistan; 3 Department of Neuroscience, Arizona State University, Arizona, USA; 3 Department of Neuroscience, Arizona State University, Arizona, USA; 4 Department of Medicine, Milton Keynes University Hospital, Milton Keynes, United Kingdom; 4 Department of Medicine, Milton Keynes University Hospital, Milton Keynes, United Kingdom; 5 Department of Medicine, Pilgrim Hospital, Boston, United Kingdom; 5 Department of Medicine, Pilgrim Hospital, Boston, United Kingdom; 6 Department of Medicine, Rawal Institute of Health Sciences, Islamabad, Pakistan; 6 Department of Medicine, Rawal Institute of Health Sciences, Islamabad, Pakistan; 7 Department of Medicine, Morriston Hospital, Swansea Bay University Health Board, Swansea, United Kingdom; 7 Department of Medicine, Morriston Hospital, Swansea Bay University Health Board, Swansea, United Kingdom; 8 Department of Family Medicine, American Hospital Dubai, Dubai, United Arab Emirates; 8 Department of Family Medicine, American Hospital Dubai, Dubai, United Arab Emirates; 9 Department of Medicine, Dubai Medical College, Dubai, United Arab Emirates; 9 Department of Medicine, Dubai Medical College, Dubai, United Arab Emirates; 10 Department of General Medicine, Gulf Medical University, Ajman, United Arab Emirates; 10 Department of General Medicine, Gulf Medical University, Ajman, United Arab Emirates; 11 Department of Internal Medicine, Sheikh Khalifa Medical City, Abu Dhabi, United Arab Emirates; 11 Department of Internal Medicine, Sheikh Khalifa Medical City, Abu Dhabi, United Arab Emirates

**Keywords:** sarcopenia, bone mineral density, chronic liver disease, osteoporosis, muscle function

## Abstract

**Introduction:**

Patients with chronic liver diseases (CLD) are likely to have low bone mineral density (BMD) and sarcopenia, complicating frailty, fractures, and death. However, this relationship is not appropriately studied among South Asian individuals.

**Objectives:**

The study aimed to establish the association between sarcopenia and BMD in CLD patients using standardised assessment tools.

**Methods:**

The proposed study is a prospective cohort study involving 501 adults with CLD in tertiary hospitals in Pakistan and collaborating international centers between December 2024 and July 2025. The baseline, 3 months and 6 months were used to determine the risk of sarcopenia and osteoporosis using the SARC-F and Osteoporosis Self-Assessment Tool for Asians (OSTA). Mann-Whitney U tests, repeated measures ANOVA and multiple linear regression were used to analyse data.

**Results:**

Men scored higher on SARC-F (p < 0.05), indicating a higher risk of sarcopenia, whereas women scored higher on OSTA (p < 0.05), indicating better bone health. Risks were also much higher at six months (F = 68.35 and 52.84, p < 0.001). Lower OSTA scores were predicted by higher SARC-F scores (B = -0.684, p < 0.001), older age, male sex, and longer disease duration, but physical activity was protective (B = 1.042, p < 0.001).

**Conclusion:**

The loss of BMD is closely associated with sarcopenia in CLD. The musculoskeletal deterioration and better outcomes in this high-risk population can be achieved through early screening and interventions to encourage physical activity.

## Introduction

Chronic liver disease (CLD) represents a significant global health burden, affecting an estimated 1.5 billion individuals worldwide^1, 2)^. Musculoskeletal disorders such as hepatic osteodystrophy, sarcopenia, and osteoporosis are common in CLD. Sarcopenia and osteoporosis are comorbid and share pathophysiology (malnutrition, inflammation, and hormonal dysregulation)^3, 4)^.

Sarcopenia, defined by a decrease in muscle mass, strength, and function, is a common condition in chronic liver disease patients that has several negative implications for health, leading to higher morbidity, metabolic disturbances, higher short-term mortality, and survival after liver transplantation^5, 6)^. It is also highly associated with frailty and fractures, for which early intervention and treatment measures are crucial^7)^.

Likewise, low bone mineral density (BMD) is frequently reported in CLD, particularly in cirrhotic patients, with the lumbar spine and forearm being the most affected sites. This predisposes individuals to osteoporosis and fractures^8, 9)^. Both sarcopenia and cirrhosis are independent risk factors for osteoporosis, and coexistence further worsens clinical outcomes in patients with CLD^10)^.

### Study gap

Patients with CLD often suffer from malnutrition, lack of rehabilitation, and late identification (of musculoskeletal complications). These effects could lead to an increased risk of early onset of sarcopenia and decreased bone mineral content, which are frequently underestimated in standard medical practice. Not only does the combination of muscle loss and poor bone quality increase the threat of falls and fractures, but it also exacerbates overall prognosis in a population already at risk. Despite the high prevalence of liver disease, the relationship between sarcopenia and bone health has not been well studied. Addressing this relationship and its implications with standardised instrumentation in a local area is necessary to present evidence on early detection, preventative measures, and more comprehensive patient care.

## Materials and Methods

### Primary objective

• To assess the relation between sarcopenia and BMD in patients with CLD by applying standardised tools.

### Secondary objectives

• To investigate the prevalence of sarcopenia among patients with CLD.

• To ascertain the prevalence of low BMD in these populations.

• To investigate demographic and clinical characteristics associated with combined sarcopenia and low bone density.

### Methodology

This study uses a prospective cohort research design to examine the correlation between sarcopenia and BMD in CLD patients. Data were collected at three time points: baseline (enrollment), 3 months, and 6 months. Baseline demographic and clinical information, including age, gender, comorbidities, and disease characteristics, was collected. The standardised scales developed to measure sarcopenia included muscle mass, muscle strength, and physical performance, while BMD was measured using validated methods. The third- and sixth-month follow-up measures allowed tracing alterations in muscle and bone health over time and discussing the correlation between these alterations in the CLD condition. This longitudinal design enabled us to compare changes within the same subjects, allowing us to understand how sarcopenia and bone loss in this population changed over time. The study did not include a control group, as its objective was to determine associations and trajectories among patients who had already been diagnosed with CLD.

The Institutional Review Board at Rawalpindi Medical University, Rawalpindi, Pakistan, approved the study under protocol number 0073f-IRB-RMU- 2024. Informed consent was obtained from all participants after they were informed about the purpose, procedures, and voluntary nature of their participation. Patients were assured of confidentiality and informed of their right to withdraw at any time without affecting their current medical treatment.

### Study setting and duration

The research took place in the hepatology and gastroenterology units of tertiary care hospitals in Pakistan and collaborating international centers. The data were gathered over six months, from December 2024 to July 2025. A purposive nonprobability sampling technique was used to recruit participants.

### Sampling

The sample size was determined using the WHO formula for sample proportion, where the proportion in the target population was estimated conservatively at 0.50, with a margin of error of 0.05 and a 95% confidence interval^11)^. According to such parameters, a sample size of 384 was needed. The number of people to be recruited was increased to account for potential dropouts and loss to follow-up within the six-month study period. Of the 550 patients with chronic liver disease initially contacted, 501 patients were included in the final analysis and had all assessments completed.

### Eligibility criteria

To be included in the study, patients must be aged 18 years or older and have established chronic liver disease from any cause and at any stage. Participants were included if they provided informed consent and had the capacity to participate in the required assessments at baseline, 3 months, and 6 months. Patients were excluded in case they had undergone liver transplantation, advanced hepatocellular carcinoma, acute liver failure, severe mobility impairment not related to liver disease, or comorbidities that are likely to impact muscle or bone health independently, including chronic kidney disease, long-term corticosteroid use, or neuromuscular disorders. Those who failed to comply with the follow-up assessment or refused to do so were also excluded.

### Data collection tools

The research employed a structured questionnaire that consisted of demographic data and two standardised measures: the SARC-F questionnaire (Strength, Assistance with walking, Rise from a chair, Climb stairs, and Falls) to determine sarcopenia, and the Osteoporosis Self-Assessment Tool (OST) to estimate the risk of low bone mineral density. Each instrument was utilised in the original English version because no validated Urdu versions were available at the time the research was conducted. Participants were assisted as needed in understanding the questions and providing accurate answers. Data were recorded at three points: baseline, three months, and six months, unless it was demographic information, in which case it was only recorded at baseline.

### Demographic information

The first part of the questionnaire collected baseline demographic and clinical data, including age, gender, education level, marital status, and employment status. The respondents were also questioned on how long they had been diagnosed with chronic liver disease and comorbid diseases, including diabetes, hypertension, and other chronic conditions, and their current medications. The data was necessary to characterise the sample and analyse possible subgroup variations in sarcopenia and bone health.

### SARC-F questionnaire

The SARC-F (Strength, Assistance with walking, Rise and a chair, Climb stairs, and Falls) is a short, self-reported instrument created by Malmstrom and Morley in 2013 to identify sarcopenia. It is divided into five items that measure each of the domains: muscle strength, functional ability, and falls history. The score is graded on a scale of 0 to 2, with 2 indicating greater functional impairment and an increased risk of sarcopenia. Thus, the scales run from 0 to 10. Scores of 4 or more are an indication that the patient has a high risk of sarcopenia^12)^.

### Osteoporosis Self-Assessment Tool (OST)

OST is an established screening tool developed by Koh et al. in 2001 to evaluate the risk of low BMD or the presence of osteoporosis based on age and body weight. It is a simple tool that estimates severity through the equation: OST Score = (Weight in kg) - (Age in years) × 0.2. The lower the score, the higher the chances of low BMD. An OST score is a single, calculated score, not composed of multiple items, making it easy to apply and interpret. The instrument has proven to be a reliable and internally consistent measure across several populations and is a popular screening tool for assessing osteoporosis risk^13)^.

### Procedure

Participants were invited to complete the structured questionnaire after signing the provided informed consent form. The questionnaire included demographic data, the SARC-F to assess sarcopenia, and the OST to estimate the risk of low BMD. The instruments were mainly self-administered; however, trained data collectors were available to assist participants who struggled to read or comprehend any items. Standardised training was provided to all data collectors to ensure they administered the questions with a uniform approach and to reduce bias in the interviewer's administration. The non-directive mode of assistance was employed to maintain neutrality and prevent the aid from having an impact. The anonymisation of responses, along with the removal of any identifying data, ensured confidentiality, allowing us to include participants from diverse educational backgrounds and health statuses.

### Statistical analysis

IBM SPSS Statistics version 26 was used to analyse data. Participants' characteristics were summarised using descriptive statistics (frequencies and percentages). Boxplots depicted the distribution of the SARC-F and OSTA scores. The Mann-Whitney U test compared gender differences, while change over time and interaction between time and age were analyzed with repeated measures of ANOVA. SARC-F and symptomatic variables were utilized to establish the predictors of osteoporosis risk (OSTA scores) by constructing a multiple linear regression model. The predicted OSTA values were demonstrated with a histogram, and statistical significance was defined as *p* < 0.05.

### Ethical guidelines

The study was conducted in accordance with the prevailing ethical norms for research involving human subjects. Special focus was placed on the rights, security, and risk of patients with chronic liver disease. Recruitment was conducted without coercion or undue influence, and the subjects were fully informed that their decision to participate or withdraw at any time would not affect their clinical care. Research practices were conducted in line with the dignity of the participants, cultural sensitivity, and respect for their autonomy. To preserve participants' privacy, confidentiality and anonymity were maintained throughout the study, and all data were stored securely.

## Results

### Description of participants' demographic and clinical profile

The demographic attributes of the 501 participants are displayed in Table 1. Most of them were between 50 and 59 years (38%), and their gender distribution was equally balanced (52% males and 48% females). The majority of the participants were married (30%), and had a secondary level of education (45%). There was an equal representation of urban and rural residents. Over half of the respondents were ex-smokers (55%), and nearly one-half had 1-3 years of liver disease (49%). The participants reported a family history of osteoporosis or fracture in 59% and a fall in the previous year in 70%. Most participants expressed a moderate level of physical activity (57%).

**Table 1:** Demographic characteristics of participants (N = 501)

Variable	f	%		Variable	f	%
Age				Smoking status		
18-29 years	71	14		Never smoked	141	28
30-39 years	40	8		Former smoker	274	55
40-49 years	149	30		Current smoker	86	17
50-59 years	189	38		Duration of liver disease		
60 years and above	52	10		< 1 year	97	19
Gender				1-3 years	243	49
Male	258	52		4-6 years	161	32
Female	243	48		Family history of Osteoporosis/Fracture		
Marital status				Yes	294	59
Single	101	20		No	207	41
Married	152	30		History of falls (past 12 months)		
Widowed	172	34		Yes	353	70
Divorced/Separated	76	15		No	148	30
Educational level				Physical activity level		
No formal education	78	16		Low	152	30
Primary	123	25		Moderate	286	57
Secondary	227	45		High	63	13
Higher (College/University)	73	14				
Residence						
Urban	250	50				
Rural	251	50				

Note. N = number of participants; f = frequency, % = percentage

### Distribution of sarcopenia and osteoporosis risk scores

**Figure 1:**
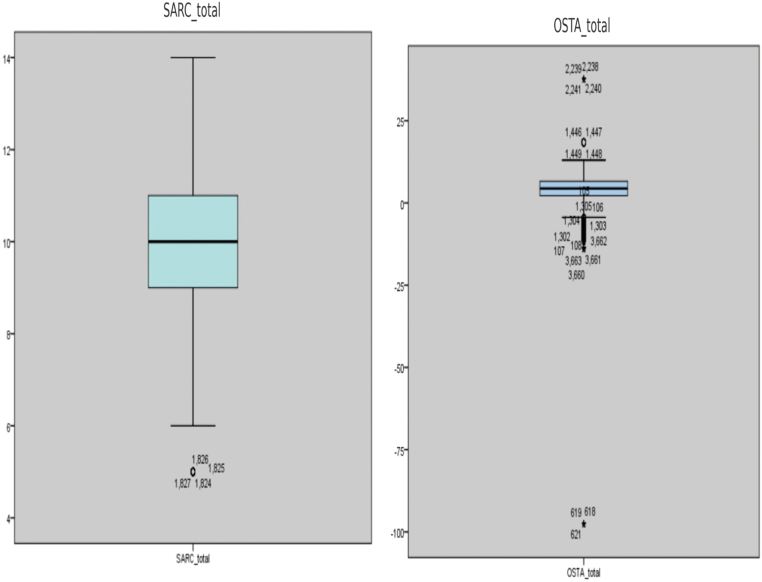
Boxplots showing the distribution of SARC-F and OSTA total scores among study participants (N = 501)

Figure 1 shows boxplots that show the distributions of the SARC-F and OSTA total scores in adults with chronic liver disease (N = 501). The SARC-F boxplot on the left illustrates the sarcopenia risk, showing moderate variability. Most values are concentrated around the median, with a few lower-end values representing low risk levels. The OSTA boxplot on the right indicates the osteoporosis risk, as the values are grouped around the median and have a reduced interquartile range. There are several outliers on the higher and lower ends, indicating participants with unusually high and low scores on OSTA. The neutral backgrounds of the two boxplots allow for better understanding and enable a visual comparison of the score distributions and the presence of outliers in the two screening tools.

### Gender-based comparison of sarcopenia and osteoporosis risk

Table 2 shows the gender-based comparisons of SARC-F and OST scores at baseline, 3 months, and 6 months using the Mann-Whitney U test. The scores of SARC-F were found to be much higher among male participants at all time points (*p* < 0.05), which means that male participants were more impaired by sarcopenia compared to their female counterparts. On the other hand, the females showed considerably high OST scores during the baseline, 3 months, and 6 months (*p* < 0.05), indicating a high probability of osteoporosis. These results suggest that there exist significant sex differences, where men are more likely to develop sarcopenia and women are more likely to develop osteoporosis with age.

**Table 2:** Gender differences in Strength, Ambulation, rising from a Chair, Stair Climbing, and History of Falling (SARC-F) and Osteoporosis Self-Assessment Tool (OST) scores at baseline, 3 months, and 6 months using Mann-Whitney U tests (N = 501)

Variable	Gender	N	Mean rank	Sum of ranks	U	Z	*p*
SARC-F (baseline)	Male	258	265.40	68,473.20	27,890.500	-2.42	0.016*
	Female	243	236.02	57,378.80			
	Total	501	-	-	-	-	-
SARC-F (3 months)	Male	258	273.85	70,653.30	25,510.000	-3.51	0.001**
	Female	243	227.02	55,198.70			
	Total	501	-	-	-	-	-
SARC-F (6 months)	Male	258	281.76	72,794.10	23,100.000	-4.73	< 0.001**
	Female	243	218.20	53,057.90			
	Total	501	-	-	-	-	-
OSTA (baseline)	Male	258	235.12	60,600.90	27,150.000	-2.10	0.036*
	Female	243	267.64	66,325.10			
	Total	501	-	-	-	-	-
OSTA (3 months)	Male	258	228.48	58,948.00	24,880.000	-2.96	0.003**
	Female	243	274.91	67,978.00			
	Total	501	-	-	-	-	-
OSTA (6 months)	Male	258	220.35	56,850.30	22,460.000	-4.12	< 0.001**
	Female	243	283.75	70,075.70			
	Total	501	-	-	-	-	-

Note. N = number of participants, SARC-F = Strength, Ambulation, rising from a Chair, Stair Climbing, and History of Falling, OST = Osteoporosis Self-Assessment Tool, U = Mann-Whitney U test; Mann-Whitney U test; *p* < 0.05, *p* < 0.01, *p* <0.001 (2-tailed) was considered statistically significant.

### Effect of age on sarcopenia and osteoporosis progression over 6 months

Table 3 illustrates that SARC-F scores rose gradually in all the age groups in the baseline to 6 months, indicating a reduction in muscle strength and mobility with time. The mean SARC-F scores were highest in participants aged ≥ 60 years (9.60 ± 1.49 at baseline to 10.85 ± 1.59 at 6 months) and lowest in participants aged 18 to 29 years (5.10 ± 1.85 to 5.85 ± 2.05). On the other hand, OST scores deteriorated across all age groups, indicating an increasing risk of osteoporosis as age advances. The oldest group (≥ 60 years) scored lowest in OST (1.38 ± 2.80 to 0.54 ± 2.20), and the youngest scored the highest (8.46 ± 2.21 to 7.72 ± 2.50). In general, the growing age correlated with a substantial increase in the risk of sarcopenia and a simultaneous decrease in bone health over 6 months.

**Table 3:** Repeated measures ANOVA and descriptive statistics showing the effect of age on Strength, Ambulation, Rising from a Chair, Stair Climbing, and History of Falling (SARC-F) and Osteoporosis Self-Assessment Tool (OST) scores over time

Age Group (years)	N	SARC (Baseline); M ± SD	SARC (3 months); M ± SD	SARC (6 Months); M ± SD	OSTA (Baseline); M ± SD	OSTA (3 Months); M ± SD	OSTA (6 Months); M ± SD
18-29 years	71	5.10 ± 1.85	5.42 ± 1.90	5.85 ± 2.05	8.46 ± 2.21	8.10 ± 2.35	7.72 ± 2.50
30-39 years	40	6.05 ± 1.75	6.45 ± 1.80	6.92 ± 1.88	4.77 ± 4.11	4.35 ± 3.98	3.92 ± 3.82
40-49 years	149	7.10 ± 1.70	7.58 ± 1.76	8.10 ± 1.84	3.65 ± 3.90	3.10 ± 3.55	2.60 ± 3.20
50-59 years	189	8.25 ± 1.65	8.80 ± 1.72	9.42 ± 1.81	2.45 ± 3.60	1.95 ± 3.25	1.42 ± 2.88
≥ 60 years	52	9.60 ± 1.49	10.18 ± 1.53	10.85 ± 1.59	1.38 ± 2.80	0.95 ± 2.45	0.54 ± 2.20

Note. N = number of participants; M = Mean, SD = Standard Deviation; Repeated-measures ANOVA was conducted to assess changes across three time points (baseline, 3 months, 6 months); SARC-F = Strength, Ambulation, rising from a Chair, Stair Climbing, and History of Falling; OST Osteoporosis Self-Assessment Tool

### Effect of time and age interaction on muscle and bone health scores

As indicated in Table 4, there was a strong overall effect of time on both SARC-F (F = 68.35, *p* < 0.001, η^2^ = 0.12) and OST scores (F = 52.84, *p* < 0.001, η^2^ = 0.095), which demonstrated that there were significant changes in muscle functioning and bone health during the study period. The strong Time x Age interactions of both SARC-F (F = 12.22, *p* < 0.001, 0.046) and OST (F = 9.18, *p* < 0.001, 0.035) indicate that the differences in changes the age brackets had experienced were significant, with older participants experiencing a bigger change in functional and bone health scores over time.

**Table 4:** Repeated measures ANOVA of time and time × Age effects on strength, Ambulation, rising from a Chair, Stair Climbing, and History of Falling (SARC-F) and Osteoporosis Self-Assessment Tool (OST) scores (N = 501)

Outcome	Effect	SS	MS	F	*p*	Partial η^2^
Strength, Ambulation, rising from a Chair, Stair Climbing, and History of Falling (SARC-F)	Time	1525.78	762.89	68.35	< 0.001**	0.12
	Time × Age	1090.64	136.33	12.22	< 0.001**	0.046
Osteoporosis Self-Assessment Tool (OST)	Time	1180.42	590.21	52.84	< 0.001**	0.095
	Time × Age	820.75	102.59	9.18	< 0.001**	0.035

Note. SS = Sum of Squares, MS = Mean Square, F = F-ratio, η^2^ = effect size; **= *p* <0.001 considered significant

### Predictors of osteoporosis risk among adults with chronic liver disease

As illustrated in Table 5, the regression model established SARC-F total score, age, sex, years of liver disease, falls history, and physical activity level as important predictors of osteoporosis risk (OSTA scores). Lower OSTA scores (B = -.684, *p* < .001) and older age (B = -1.128, *p* < .001) were significantly related to higher SARC-F scores. Reduced OSTA scores were also predicted by male gender (B = -.476, p = 0.001), longer disease duration (B = -.532, p = 0.001) and history of falls (B = -.348, p = 0.002). On the other hand, OSTA scores were positively linked to higher levels of physical activity (B = 1.042, p < 0.001), indicating that physical activity helps in preventing osteoporosis. On the whole, the model indicates that the severity of sarcopenia, the progression of age, male sex, long-lasting liver disease, and falls are factors that increase the risk of osteoporosis, while physical activity reduces it.

**Table 5:** Multiple linear regression predicting osteoporosis risk (OSTA Scores) from SARC-F and clinical characteristics among adults with chronic liver disease

Predictor	B	SE	β	t	*p*	95% Confidence Interval for B LL	95% Confidence Interval for B UL
Constant (OSTA)	8.762	0.462	-	18.962	< 0.001**	7.856	9.668
SARC-F total	-0.684	0.029	-0.621	-23.586	< 0.001**	-0.741	-0.627
Age	-1.128	0.043	-0.285	-26.233	< 0.001**	-1.212	-1.044
Gender (1 = Male, 0 = Female)	-0.476	0.097	-0.042	-4.907	< 0.001**	-0.667	-0.285
Duration of liver disease (years)	-0.532	0.071	-0.071	-7.493	< 0.001**	-0.672	-0.392
History of falls (past 12 months)	-0.348	0.113	-0.025	-3.079	0.002**	-0.569	-0.127
Physical activity level	1.042	0.082	0.096	12.707	< 0.001**	0.882	1.202

Note. This table presents the results of multiple linear regression analyses predicting changes in Osteoporosis Risk (OSTA Scores); Independent variables included Strength, Ambulation, rising from a Chair, Stair Climbing and History of Falling (SARC-F) total scores, age, gender, duration of liver disease, history of falls, and physical activity level; p values < 0.01, < 0.001 indicate statistical significance.

### Normal distribution of predicted osteoporosis risk scores

**Figure 2:**
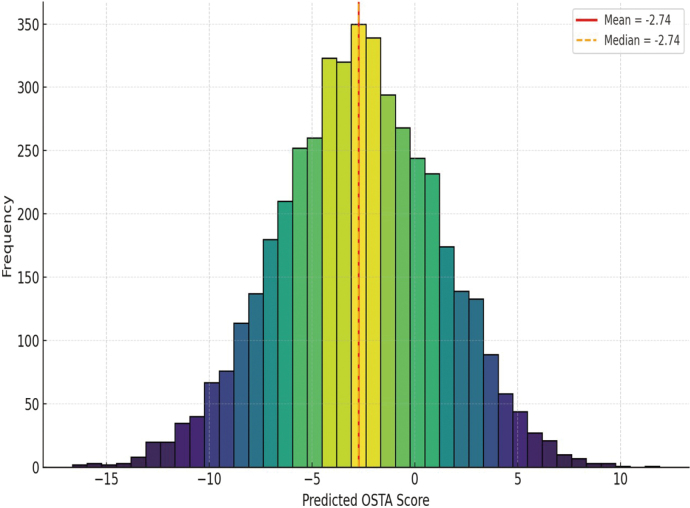
Histogram depicting the distribution of predicted OSTA scores based on the multiple regression model

Figure 2 shows a histogram of the distribution of the predicted OSTA scores from the multiple regression model. The distribution is approximately normal and symmetric, with the majority of expected scores closer to the mean and median value of -2.74. The bell-shaped curve implies that most of the predictions generated by the regression model were concentrated around the mean risk level, with fewer participants on the left and right extremes. This trend indicates a fitting data model, as the OSTA scores predicted by the model follow a near-normal distribution with no significant skewness or outliers.

## Discussion

This prospective cohort study investigated the relationship between sarcopenia and bone mineral density (BMD) in patients with chronic liver disease (CLD) using the standardised SARC-F and OSTA scales. The current research showed that at the baseline and follow-ups, the male participants consistently scored higher in SARC-F in comparison to females, which is indicative of an increased risk of sarcopenia and slower functional recovery. This trend is consistent with the results of previous community-based studies, in which men had a greater incidence of confirmed sarcopenia and worse muscle performance despite similar or even greater muscle mass. These researchers indicate that there is a possibility of an accelerated deterioration of physical functioning among men with age, which might be due to variations in muscle quality, hormones, or activity levels^14)^. Our study revealed that females scored higher on OSTA than males at baseline, 3 months, and 6 months, suggesting that females had better bone health over time. A previous community-based study supports this, showing that females had higher OSTA values than males, demonstrating the relevance of OSTA in determining gender-based differences in osteoporosis risk^15)^.

The SARC-F scores remained relatively stable across age and time, suggesting persistent or progressive sarcopenic symptoms in older adults. Similarly, with increasing age, OSTA scores decreased, indicating that participants were at increased risk of osteoporosis. This finding is consistent with the literature, which shows that both sexes experience age-related bone weakening due to hormonal reduction and the aging process^16, 17)^.

In our regression analysis, the higher sarcopenia risk represented by higher SARC-F scores was strongly connected with lower OSTA scores, which represented poorer bone health and increased risk of osteoporosis. This is in line with the idea of osteosarcopenia, where sarcopenia and osteoporosis coexist and share similar risk factors, resulting in frailty and poor clinical outcomes^18)^. In our regression analysis, we found that age was significantly correlated with low OSTA scores, indicating deteriorating bone health. This is in line with the literature that calls osteoporosis an age-related disorder whereby bone mass continues to deplete in both men and women as they grow older^17)^. Additionally, females scored higher on OSTA than males across all time frames, reflecting improved bone health. This coincides with previous research, which also found that females had high OSTA scores, confirming the tool's effectiveness in detecting gender variation in the risk of osteoporosis^15)^.

Our findings that long-term CLD is associated with lower OSTA scores align with prior research indicating that the risk of osteoporosis is elevated in patients with long-term liver disease. We consider it essential to monitor bone health regularly in such patients^19)^. The result of lower OSTA scores in participants with a history of falls is consistent with the past literature, indicating that fall history alone is a stronger predictor of increased fracture risk, which supports the significance of fall history in osteoporosis evaluation^20)^. Conversely, higher physical activity levels were significantly associated with higher OSTA scores, indicating better bone health. This corresponds to past findings of how physical activity, particularly resistance and multi-type exercises, enhances bone mineral density and prevents the occurrence of osteoporosis in older people^21)^.

In general, our results indicate that sarcopenia and osteoporosis are closely related in patients with CLD and that multimodal screening and treatment strategies should be implemented to address both muscle and bone health.

### Clinical implications

These findings have direct clinical implications for hepatology and rehabilitation practice. Routine screening for sarcopenia and osteoporosis should be integrated into the standard management of chronic liver disease, using simple tools such as SARC-F and OSTA during outpatient visits. Early identification of high-risk individuals can allow hepatologists to initiate timely referrals to rehabilitation services, while physiotherapists can design individualized exercise and mobility programs to prevent frailty and improve functional outcomes.

In addition, preventive and therapeutic interventions targeting both muscle and bone health should be emphasized. Evidence supports the role of progressive resistance training, adequate dietary protein intake, vitamin D and calcium supplementation, and supervised physiotherapy in improving musculoskeletal outcomes among CLD patients. A multidisciplinary approach involving hepatologists, dietitians, and rehabilitation specialists can optimize patient care and reduce the risk of falls, fractures, and disability.

### Limitations and future directions

Several limitations should be noted. To begin with, the research utilised screening tools (SARC-F and OSTA) rather than objective tests, including dual-energy X-ray absorptiometry (DXA) and handgrip dynamometry. Even though both scales are validated, both might miss subclinical muscle and bone deficits. Second, the assessments were all in English, which may have created comprehension problems for participants who were not highly literate, even though they were assisted. Third, because it is a self-reported, observational design, causal inference is weak, and reverse causation between sarcopenia and bone loss is possible. Fourth, potential confounders, including dietary intake, indices of liver disease severity (e.g., Child Pugh or MELD score), and vitamin D levels, were not controlled, which could have affected both sarcopenia and bone health outcomes.

Further studies are needed to confirm and elaborate these results by using more specific diagnostic and research instruments, such as DXA and computed tomography, to determine the muscle mass. Interventional studies measuring the impact of customised exercise, nutritional supplementation, or pharmacological interventions on muscle and bone parameters in CLD patients would help deepen understanding of prevention and management interventions. Also, Urdu-language versions of the SARC-F and OSTA scales should be developed and validated to improve accessibility and accuracy in local populations.

## Conclusion

Overall, the current research indicates that there is a significant progressive interconnection between sarcopenia and reduced bone mineral density in patients with chronic liver disease. The results show that older age, male gender, longer disease duration, and a history of falls are significant risk factors, whereas increased physical activity is a protective factor. The findings indicate the necessity of early and combined screening of sarcopenia and osteoporosis in patients with CLD to minimize the risk of frailty, fractures, and functional impairment later. Multidisciplinary care techniques, including nutritional optimization, physiotherapy, and exercise-based interventions, can enhance the musculoskeletal system and improve the quality of life in this susceptible population.

## Author contributions

YM conceived and designed the study and supervised the overall project. MO contributed to the study design, patient assessments, and data interpretation. KK performed statistical analysis and drafted the results. MMK assisted in the literature review, data validation, and figure preparation. HW contributed to project coordination and manuscript drafting. MGH participated in patient recruitment and data collection. MZ drafted the manuscript, coordinated revisions, and served as the corresponding author. KMH contributed to data analysis and critical review of the methodology. IWS assisted in clinical interpretation and review of discussion outcomes. SLCM performed reference management and assisted in manuscript formatting. MSAH contributed to data entry and verification of tables and figures. EEM critically reviewed the final manuscript and provided expert input on internal medicine aspects. All authors read and approved the final manuscript.

## Conflicts of interest statement

The authors declare that there are no conflicts of interest.
